# Quantification and Localization of Intracellular Free Mg^2+^ in Bovine Chromaffin Cells

**DOI:** 10.1155/MBD.2002.69

**Published:** 2002

**Authors:** Liliana P. Montezinho, Carla P. Fonseca, Carlos F. G. C. Geraldes, M. Margarida C. A. Castro

**Affiliations:** 1 Department of Biochemistry University of Coimbra P.O. Box 3126 Coimbra 3001-401 Portugal; 2 Center for Neuroscience and Cell Biology University of Coimbra P.O. Box 3126 Coimbra 3001-401 Portugal

## Abstract

Magnesium is an essential element for all living systems. The quantification of free intracellular Mg^2+^ concentration ([Mg^2+^]_i_) is of utmost importance since changes in its basal value may be an indication of different pathologies due to abnormalities of Mg^2+^ metabolism. In this work we used ^31^P NMR and fluorescence spectroscopy to determine the resting [Mg^2+^]_i_ in bovine chromaffin cells, a neuron-like cellular model, as well as confocal laser scanning microscopy to study the free Mg^2+^ spatial distribution in these cells. ^31^P NMR spectroscopy did not prove to be effective for the determination of [Mg^2+^]_i_ in this particular case due to some special morphological and physiological properties of this cell type. A basal [Mg^2+^]_i_ value of 0.551 ± 0.008 mM was found for these cells using fluorescence spectroscopy and the Mg_2+_-sensitive probe furaptra; this value falls in the concentration range reported in the literature for neurons from different sources. This technique proved to be an accurate and sensitive tool to determine the [Mg^2+^]_i_.

lntraceilular free Mg^2+^ seems to be essentially localized in the nucleus and around it, as shown by confocal microscopy with the Mg^2+^-sensitive probe Magnesium Green. It was not possible to derive any conclusion about free Mg^2+^ localization inside the chromaffin granules and/or in the cytoplasm due to the lack of sufficient spatial resolution and to probe compartmentalization.


### Quantification and Localization of Intracellular Free $Mg^{2+}$ in Bovine Chromaffin Cells

Liliana P. Montezinho[1,2], Carla P. Fonseca[1,2], Carlos F. G. C. Geraldes[1,2] and M. Margarida C.A. Castro[1,2,*]

[1]*Department of Biochemistry,* [2]*Center for Neuroscience and Cell Biology, University of Coimbra, P.O. Box 3126, 3001-401 Coimbra, Portugal*

### ABSTRACT

Magnesium is an essential element for all living systems. The quantification of free intracellular $Mg^{2+}$ concentration ($[Mg^{2+}]_i$) is of utmost importance since changes in its basal value may be an indication of different pathologies due to abnormalities of $Mg^{2+}$ metabolism. In this work we used $^{31}P$ NMR and fluorescence spectroscopy to determine the resting $[Mg^{2+}]_i$ in bovine chromaffin cells, a neuron-like cellular model, as well as confocal laser scanning microscopy to study the free $Mg^{2+}$ spatial distribution in these cells. $^{31}P$ NMR spectroscopy did not prove to be effective for the determination of $[Mg^{2+}]_i$ in this particular case due to some special morphological and physiological properties of this cell type. A basal $[Mg^{2+}]_i$ value of $0.551 \pm 0.008$ mM was found for these cells using fluorescence spectroscopy and the $Mg^{2+}$-sensitive probe furaptra; this value falls in the concentration range reported in the literature for neurons from different sources. This technique proved to be an accurate and sensitive tool to determine the $[Mg^{2+}]_i$.

Intracellular free $Mg^{2+}$ seems to be essentially localized in the nucleus and around it, as shown by confocal microscopy with the $Mg^{2+}$-sensitive probe Magnesium Green. It was not possible to derive any conclusion about free $Mg^{2+}$ localization inside the chromaffin granules and/or in the cytoplasm due to the lack of sufficient spatial resolution and to probe compartmentalization.

### INTRODUCTION

The importance of the extracellular and intracellular magnesium ion ($Mg^{2+}$) has become gradually recognized during the last century. Nowadays it is well known that an altered metabolism of $Mg^{2+}$ is involved in several pathologies. This cation is essential for all living systems, being the most abundant in tissues,

* Corresponding author: M. Margarida C.A. Castro, Department of Biochemistry, Faculty of Sciences and Technology, University of Coimbra, P.O. Box 3126, 3001-401 Coimbra, Portugal

Tel: 00-351-239-853609; Fax: 00-351-239-853607; Email: gcastro@ci.uc.pt exceeded only by the potassium ion ($K^+$) /1/. $Mg^{2+}$ plays a vital role in several cellular regulatory processes, but it is unlikely that it can have a "trigger" function like the calcium ion ($Ca^{2+}$) does, due to its concentrations inside and outside the cells, and due to its chemical properties. However, slow and small magnitude changes in $Mg^{2+}$ concentration can be important in the fine control and coordination of cellular activity /2/. $Mg^{2+}$ is involved in the synthesis of DNA and RNA, as well as in the maintenance of their conformation /3/. This cation has also been implicated in the control of membrane fluidity and permeability /4, 5/ and has been directly associated with the secretion of hormones, like insulin /6/ and prolactin /7/. Many of the actions of $Mg^{2+}$ result from its role as a co-factor of a wide range of enzymes, in the form of Mg-ATP/ADP or Mg-GTP/GDP complexes /2/. $Mg^{2+}$ activates virtually all the enzymes involved in the metabolism of phosphorylated compounds, as well as many enzymes of the glycolytic and tricarboxylic acid pathways /8/. Additionally, $Mg^{2+}$ is necessary for the optimal performance of the $Na^+/K^+$-ATPase and $Ca^{2+}$ pump /9/ and it affects different transport systems, such as the $Na^+/K^+/Cl^-$ co-transport, the $Na^+/H^+$ and $Cl^-/HCO_3^-$ exchange /10/ and several ion channels /11/. To help understand the role of intracellular $Mg^{2+}$, its homeostasis and regulation, accurate measurements of total and free intracellular magnesium concentrations ($[Mg^{2+}]_i$) were developed. The total intracellular magnesium concentration ($[Mg^{2+}]_T$) can be determined by atomic absorption spectrophotometry (AAS), inductively-coupled plasma atomic emission spectrophotometry, thin layer and ion chromatographies /12, 13, 14/. These methods require submitting the sample to several destructive steps before measuring the $[Mg^{2+}]_T$, which implies that there may be a source of sample contamination. Also, the invasive nature of these techniques may lead to errors in the study of $Mg^{2+}$ transport due to non-specific binding of $Mg^{2+}$ to cell membranes and other components, as well as additional ion transport during sample preparation /15/. However several methods can be used to determine $[Mg^{2+}]_i$ in intact cells, such as $Mg^{2+}$-sensitive microelectrodes, nuclear magnetic resonance (NMR) spectroscopy, fluorescence techniques and coupled assays using $Mg^{2+}$-dependent enzymes /14, 16, 17, 18, 19/.

The improvement of these methods to determine both total and free intracellular $Mg^{2+}$ concentrations indicates that only 5-10% of the total $Mg^{2+}$ is free, the remaining being bound to highly charged anionic ligands such as ATP, ADP, RNA, polyphosphates, proteins and citrate /20,21/. A substantial amount of the total $Mg^{2+}$ is distributed in the nucleus, endoplasmic reticulum, mitochondria and cytoplasm /16/.The basal $[Mg^{2+}]_i$ varies significantly from tissue to tissue: 0.6- 1.5 mM for striated muscle /22, 23, 24/, 0.2-0.5 mM for smooth muscle /23, 25/, 0.8 mM in perfused rat hearts /26, 27/, 0.3 mM in synaptosomes /28/, 0.37 mM in hepatocytes /29/ and 0.5 - 1.2 mM in cardiac cells /30/, 0.8 mM for cultured rat forebrain neurons /31/, $0.63 \pm 0.03$ mM for cultured rat brain cortex neurons /32/, 0.4-0.7 mM for rat liver /33, 34/. A $[Mg^{2+}]_i$ value of 0.3 mM has been reported for human brain /35/ and 0.58 - 1.0 mM for rat brain /24, 33, 36/. Changes in the basal intracellular free $Mg^{2+}$ concentration are an indication of abnormalities of $Mg^{2+}$ metabolism, which are correlated with different pathologies such as diabetes, hypertension and dyslipidaemia /37, 38/. Therefore it is of utmost importance to determine intracellular free $Mg^{2+}$ concentration, $[Mg^{2+}]_i$, because this measurement may contribute as a diagnostic tool of some diseases.

Bovine chromaffin cells, obtained from bovine adrenal medulla, are a good neuronal model because they are endocrine and sympathetic neuron-like cells, originated from the same precursor cells as those of the sympathetic ganglion cells, and constitute a convenient model to study a variety of neurological disorders

/39,40/. These cells have a higher degree of intracellular complexity when compared with other types of cells; they contain chromaffin granules, which are highly heterogeneous organelles with an unusually high ionic strength and low pH value (~5.5 /41/), a high concentration of ATP complexed with catecholamines, chromogranin A and some ions /42/.

In this work we used two different techniques to determine $[Mg^{2+}]_i$ ($^{31}P$ NMR and fluorescence spectroscopies) in bovine chromaffin cells, and another one to study the spatial distribution of $Mg^{2+}$ inside these cells (confocal microscopy). The advantages and limitations of each method for measuring $[Mg^{2+}]_i$ are presented and discussed in this communication.

### MATERIALS AND METHODS

### Materials

Furaptra (salt form), furaptra-AM (cell permeant acetoxymethyl (AM) ester form), Magnesium Green-AM™ (cell permeant acetoxymethyl ester form) and Pluronic® F-127 were obtained from Molecular Probes (Leiden, The Netherlands). Collagenase (type B) was purchased from Boehringer Mannheim (Mannheim, Germany). Percoll was supplied by Pharmacia Biotech AB (Uppsala, Sweden), fetal calf serum (FCS) by Seromed Biochrom (Berlin, Germany), Dulbecco's Modified Eagle's Medium / Ham's Nutrient Mixture F-12 (DMEM/F-12, 1:1 mixture) from GibcoBRL, Life Technologies (Gaithersburg, MD, USA), Bovine Serum Albumin (BSA), Trypan Blue; Neutral Red, antibiotic antimycotic, N-2-hydroxyethylpiperazine-N′-2-ethanesulfonic acid (HEPES), ethylene glycol-bis(2-aminoethylether)- N, N, N′, N′- tetraacetic acid (EGTA), Poly-L-lysine, $NaHCO_3$, NaCl, glucose, and low-gelling temperature agarose were purchased from Sigma Chemical Company (St. Louis, MO, USA), KCl, $CaCl_2$, and $MgCl_2$ from Merck (Darmstadt, Germany) and Urografin® from Schoering AG (Berlin, Germany).

### Isolation and culture of bovine chromaffin cells

Bovine adrenal glands were obtained from a local slaughterhouse and transported to the laboratory on ice. Bovine chromaffin cells were isolated from bovine adrenal glands and purified on a Percoll gradient as previously described /43/. Cells were then cultured in a 1:1 mixture DMEM/F-12 (1.56%) medium with 15 mM HEPES and 26 mM $NaHCO_3$, supplemented with 5% of heat-inactivated fetal calf serum, penicillin (100 units/mL), streptomycin (100 μg/mL) and amphotericin B (0.25 μg/mL), at 37 °C, in a humidified $CO_2$ (5%) and air (95%) atmosphere.

The cells were cultured up to a density of 1 million cells/mL in 100 mm Petri dishes and maintained in culture for 3 days before the NMR experiments.

For the fluorescence experiments, the cell preparation was further purified by a Urografin gradient as described before /44, 45/ and were used 2 days after plating. The cells were plated at a density of 0.8 million cells/$cm^2$ on square cover-slips (1$cm^2$) previously coated with poly-L-lysine when fluorescence spectroscopy was used, and at a density of 0.15 million cells/mL/well on glass cover-slips (16 mm diameter), also coated with poly-L-lysine, in 12-well plates for confocal microscopy experiments.

### $^{31}P$ NMR spectroscopy

*i) NMR sample preparation*

After 3 days in culture, bovine chromaffin cells were resuspended and centrifuged at 115 *g* (800 rpm) during 8 min at 25 °C (Sigma 3K10). The pellet was resuspended in culture medium up to a volume of 500 µL.

Bovine chromaffin cells were immobilized in agarose gel threads, placed in a 10 mm NMR tube and perfused with oxygenated culture medium (5% $CO_2$ / 95 % $O_2$), at 37 °C, pH 7.35. The immobilization was performed by mixing the 500 µL of cell suspension (50 to 75 million cells) with 500 µL of Krebs medium (in mM: NaCl 140, KCl 5, $CaCl_2$ 2, $MgCl_2$ 1, glucose 10, HEPES 20, pH 7.35) containing 2% of low-gelling temperature agarose, in a 1:1 proportion (final agarose concentration of 1%), at 37 °C. The threads were formed by passing this cell mixture through a Teflon tubing with 0.5 mm internal diameter, partially submersed in ice. Once it was passed through the iced portion of the tubing, the mixture solidified and threads with 0.5 mm diameter with entrapped cells were formed into the 10 mm NMR tube. The immobilized cells were continuously perfused at approximately 1 mL/ min with culture medium, pH 7.35. This procedure ensures the maintenance of cell viability throughout the NMR experiments, as already demonstrated with another type of cells /46, 47/ and with chromaffin cells in our lab (data not shown).

*ii) NMR experiments*

Proton decoupled $^{31}P$ NMR spectra were acquired at 202.3 MHz, 37 °C on a Varian Unity-500 NMR spectrometer equipped with a multinuclear 10 mm broad-band probe and a controlled temperature unit. The acquisition parameters were as follows: 1200 transients, spectral width of 14998 Hz, pulse width of 18 µs, interpulse delay of 0.55 s, acquisition time of 0.996 s and line broadening of 30 Hz. $H_3PO_4$ 85% was used as an external reference at 0 ppm.

*iii) Measurement of intracellular free $Mg^{2+}$ concentration by $^{31}P$ NMR*

To calculate the $[Mg^{2+}]_i$ from the $^{31}P$ NMR data, the following equations were used /48, 49/:

$$[Mg^{2+}]_i = (K_b)^{-1}((1/X_f)-1) \quad (1)$$

$$X_f = [ATP]_f / [ATP]_T = (\Delta\delta_{\alpha\beta}^{obs} - \Delta\delta_{\alpha\beta}^{b})/(\Delta\delta_{\alpha\beta}^{f} - \Delta\delta_{\alpha\beta}^{b}) \quad (2)$$

where $\Delta\delta_{\alpha\beta}^{f}$, $\Delta\delta_{\alpha\beta}^{b}$ and $\Delta\delta_{\alpha\beta}^{obs}$ are the chemical shift differences between the resonances of α and β phosphates of ATP in the absence of $Mg^{2+}$, in the presence of saturating amounts of $Mg^{2+}$, and the one observed for a given sample, respectively. The $\Delta\delta_{\alpha\beta}^{f}$, $\Delta\delta_{\alpha\beta}^{b}$ values obtained in cell-free solutions of ATP are respectively 10.82, 8.43 ppm /50, 51/ and $X_f$ is the mole fractions of all the ATP species not chelated to

$Mg^{2+}$. $[ATP]_T$ is the concentration of total ATP and $[ATP]_f$ the concentration of all ATP not bound to $Mg^{2+}$. The value of $K_b$, the binding constant of $Mg^{2+}$-ATP, is 25,000 $M^{-1}$ at 37 °C and pH 7.3 /52/.

### Fluorescence Techniques

*i) Fluorescence spectroscopy experiments*

The fluorescence data were obtained on a SPEX FluoroMax Fluorimeter, at 30°C, using furaptra, the $Mg^{2+}$ specific fluorescent probe. This indicator binds to $Mg^{2+}$ resulting in an excitation blue shift in the spectrum from 335 nm to 370 nm with increasing amounts of $Mg^{2+}$. Its chemical properties are insensitive to pH and $Ca^{2+}$ levels in the physiological range [16, 53]. The $[Mg^{2+}]_i$ was monitored using the cell-permeant acetoxymethyl ester form of furaptra (furaptra-AM). Bovine chromaffin cells adherent to 1cmx1cm square cover-slips (0,8x$10^6$ cells/$cm^2$) were incubated, for 45 minutes, in a humidified $CO_2$ (5%) and air (95%) atmosphere, at 37 °C, in a Krebs medium containing 1% BSA, 5 μM of furaptra-AM and 0.1% Pluronic® F-127, previously sonicated for 3 minutes. After loading the cells with the fluorescent probe, they were incubated for an additional period of 20 minutes in Krebs medium containing 1% BSA and then washed with a Krebs medium containing 0.2% BSA. The cover-slips containing the furaptra-loaded cells (previously washed in Krebs medium) were then attached to plastic holders and placed in a fluorescence cuvette containing 1.5 mL of Krebs medium.

The emission wavelength was fixed at 500 nm, and the excitation wavelength changed between 300 and 400 nm (5 nm emission and excitation slits). The fluorescence intensity ratio R ($F_{335}/F_{370}$) values were taken immediately after replacing the Krebs medium by a fresh one (every 15 minutes during 2h 15min) in order to remove any released fluorescent probe from the cells to the extracellular medium that could contribute to an over-estimation of the R(($F_{335}/F_{370}$) value.

*ii) Calculation of $[Mg^{2}\ ]_i$ values from fluorescence spectroscopy data*

When using furaptra, the $[Mg^{2+}]_i$ was determined by direct application of equation (3) /16, 53/:

$$[Mg^{2+}]_i = K_d (R - R_{min})/(R_{max} - R)\ S_{min}/S_{max} \quad (3)$$

where $K_d$ is the dissociation constant of the furaptra/$Mg^{2+}$ complex (1.5 mM at 37 °C; /16/), R, $R_{min}$ and $R_{max}$ are the fluorescence intensity ratios at 335 nm and 370 nm obtained for the experimental sample at different time points, in the absence and in the presence of saturating amounts of $Mg^{2+}$, respectively. $S_{min}$ and $S_{max}$ are the fluorescence intensities at 370 nm in the absence and in the presence of saturating amounts of $Mg^{2+}$, respectively. The $R_{min}$ and $S_{min}$ parameters were determined in a $Mg^{2+}$-free and $Ca^{2+}$-free solution (in mM: KCl 120, NaCl 20, HEPES 10, EGTA 1, pH 7.35) containing 2 μM of furaptra (salt form). The $R_{max}$ and $S_{max}$ parameters were determined by adding the salt form of furaptra (2 μM) to a $Mg^{2+}$-saturated solution (in mM: $MgCl_2$ 70, KCl 15, NaCl 20, HEPES 10, pH 7.35) /54/.

*iii) Confocal microscopy experiments*

The spatial distribution of intracellular free magnesium ion ($Mg^{2+}{}_i$) was observed using the $Mg^{2+}$ specific fluorescent probe Magnesium Green, instead of furaptra, because it has the physical properties adequate to the technical characteristics of the available confocal equipment. This indicator exhibits a higher affinity for $Mg^{2+}$ ($K_d$ ~1.0 mM) than does furaptra ($K_d$ ~1.9 mM) or mag-indo-1 ($K_d$ ~2.7 mM); this indicator also binds $Ca^{2+}$ with moderate affinity ($K_d$ for $Ca^{2+}$ in the absence of $Mg^{2+}$ ~6 μM), at 22°C /55/. Upon binding $Mg^{2+}$, Magnesium Green exhibits an increase in fluorescence emission intensity without a shift in wavelength /55/. We used a MRC600 confocal imaging system (BioRad laboratories, Milan, Italy) linked to a Nikon Optiphot-2 fluorescence microscope. A krypton/argon mixed laser was used in combination with a 488 nm band pass filter (excitation) and a 585 long-pass filter (emission). The cells, adherent to coverslips, were incubated with Krebs medium supplemented with 1% BSA and 5 μM of the cell-permeant acetoxymethyl ester form of this $Mg^{2+}$ indicator (Magnesium Green-AM™) during 45 min, in a humidified $CO_2$ (5%) and air (95%) atmosphere, at 37 °C. After this loading period, the medium was replaced by fresh Krebs medium containing 1% BSA and the cells were incubated for an additional period of 15 min, under the same atmosphere conditions. Then the coverslips with the attached cells were washed three times with a Krebs medium containing 0.2% BSA and placed in a special perfusion camera in the confocal apparatus. This camera was specially made in order to allow the continuous perfusion of the cells with Krebs medium, at 37 °C, during the time course of the confocal microscopy experiments, ensuring the maintenance of cellular viability. The flux rate of the medium surrounding the cells inside the camera was set to 1 mL/min with a peristaltic pump (MasterFlex®, Cole-Parmer Instrument Co., Illinois, USA). The images were acquired with the excitation and emission wavelengths fixed to 488 nm and 585 nm, respectively. Fluorescence images were treated using confocal assistant and PainShopPro softwares.

### RESULTS AND DISCUSSION

### $^{31}P$ NMR experiments

$^{31}P$ NMR spectroscopy was used in order to determine the $[Mg^{2+}]_i$ and also to check the cell viability. The spectra were acquired over time from chromaffin cells immobilised in agarose gel threads and perfused with the oxygenated DMEM/F-12 medium (as described in the Methods section) (**Fig. 1**).

The $^{31}P$ NMR chemical shift difference between the $P_\alpha$ and $P_\beta$ NMR resonances of ATP ($\Delta\delta_{\alpha\beta}$) is sensitive to the amount of $Mg^{2+}$ bound to ATP /48, 49/. In water and in the absence of other ions it varies from a maximum value in the absence of free $Mg^{2+}$ (10.82 ppm /50,51/) to a minimum value in the presence of saturating amounts of $Mg^{2+}$ (8.43 ppm /50,51/). Using equations (1) and (2), $[Mg^{2+}]_i$ can be calculated through the $\Delta\delta_{\alpha\beta}$ value observed for the biological sample. As already described, there are two different pools of ATP in chromaffin cells /56/: the cytosolic ($ATP_{cyt}$) and the granular ($ATP_{gran}$) pools. Vesicular ATP is distinguishable from cytosolic ATP because it is sequestered with catecholamines, chromogranin A, $Ca^{2+}$ and other ions in lower concentrations. The ATP from these two different pools is in slow exchange conditions in the $^{31}P$ NMR time scale, giving rise to differentiated resonances for $ATP_{cyt}$ and $ATP_{gran}$. As already described

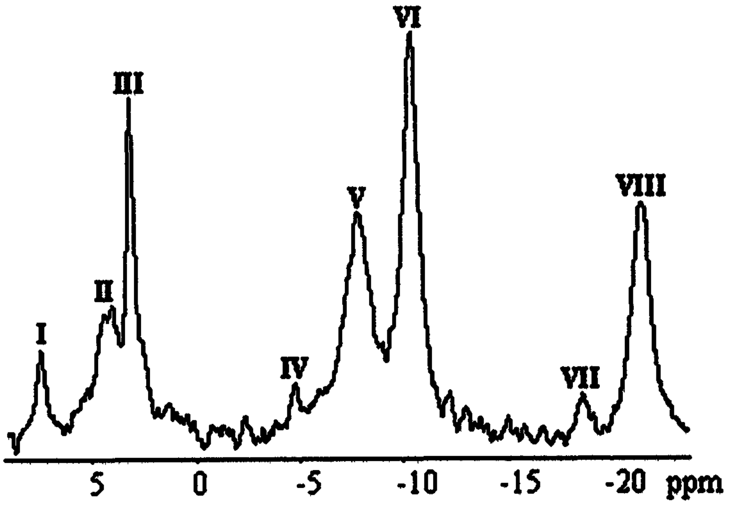

**Fig. 1:** $^{31}P$ NMR spectrum, obtained at 202.3 MHz, of bovine chromaffin cells immobilized in agarose gel threads and perfused with oxygenated DMEM/F-12 (1:1) medium, at 37 °C. Phosphoric acid 85% was used as external reference at 0 ppm. The assignments are as follows: I – Phosphomonoesters (PME); II – Sugar phosphates; III – Inorganic phosphate ($P_i$); IV – Cytosolic $P_\gamma$-ATP; V – Granular $P_\gamma$-ATP; VI – Granular $P_\alpha$-ATP; VII – Cytosolic $P_\beta$-ATP; VIII – Granular $P_\beta$-ATP.

in the literature /57/, the $P_\alpha$ resonance of $ATP_{cyt}$ is hidden under the $P_\alpha$ signal of $ATP_{gran}$, preventing an accurate measurement of $\Delta\delta_{\alpha\beta}$ of $ATP_{cyt}$, thus not allowing the exact determination of free intracellular $Mg^{2+}$ concentration in the cytosol, using equations (1) and (2). Moreover, as the internal pH of the vesicles is low (when compared with the pH value of other intracellular organelles) and because of the association of ATP with the positively charged catecholamines within the granules, the $P_\alpha$ and $P_\beta$ NMR chemical shifts of $ATP_{gran}$ are unusual /58, 59/. In consequence $\Delta\delta_{\alpha\beta}$ is higher in the chromaffin vesicle (the value obtained by us was 11.08 ± 0.02 ppm (n=4)) than the theoretically expected value for ATP in the absence of $Mg^{2+}$ (10.82 ppm, /50, 51/), leading to negative $[Mg^{2+}]_i$ values when equations (1) and (2) are used.

Therefore, one may conclude that this technique, which has been widely used with biomolecules /50, 60, 61, 62/ and other cell types /47, 63/ to determine $[Mg^{2+}]_i$ concentrations, is not applicable to chromaffin cells due to their particular characteristics. In fact, ATP compartmentalization and overlap of signals prevent the use of this technique to determine $[Mg^{2+}]_i$ in the cytosol, whereas the high ionic strength and specific ATP interactions in the granule make the use of the aqueous model $\Delta\delta_{\alpha\beta}$ values in equation (2) inadequate.

The cells were viable under these experimental conditions for several hours, as demonstrated by the maintenance of the energetic cellular levels (determined by the ratio of the NMR signal integrals $(\beta\text{-ATP})_{cyt}$/ $(P_i)_{cyt}$) /64/ and the visibility of the cytosolic $P_\gamma$ and $P_\beta$ ATP NMR signals /57/, and of the intragranular pH (5.50 ± 0.02, n=3) calculated from the chemical shift value of $P_\gamma$-$ATP_{gran}$ NMR signal using the calibration curve determined by Njus *et al.* /58/, which is in agreement with the average granular pH value (5.65 ± 0.15) in "resting" chromaffin granules /58/.

### Fluorescence spectroscopy experiments

Fluorescence spectroscopy and the $Mg^{2+}$ fluorescent probe furaptra were used to determine the basal intracellular $[Mg^{2+}]_i$ in chromaffin cells. These studies were carried out with the cells adherent to coverslips, which allowed us not only to ensure the viability of the cells, but also to remove any fluorescent probe released from the cells with time, which could affect the observed fluorescence intensity ratio value, $R(F_{335}/_{370})$, inside the cells.

The R values were converted into $[Mg^{2+}]_i$ using equation (3). It was observed that the basal $[Mg^{2+}]_i$ was maintained constant over the experimental time course (2h 15min), with a mean value of 0.551 ± 0.008 mM (n=12). No similar study was reported in the literature for this cell type. As these cells are neuron-like, a comparison of the basal $[Mg^{2+}]_i$ in neurons is pertinent; actually the value obtained in this study falls into the concentration range reported in the literature for neurons from different sources: 0.8 mM for cultured rat forebrain neurons /31/, 0.63 ± 0.03 mM for cultured rat brain cortex neurons /32/, 0.3 mM for human brain /35/, 0.58 - 1.0 mM for rat brain /24, 33, 36/. The experimental value of 0.551 ± 0.008 mM for $[Mg^{2+}]_i$ in bovine chromaffin cells is also in agreement with the observation that only 5-10% of the total intracellular $Mg^{2+}$ is free /20, 21/, being the total $Mg^{2+}$ content value of 11.5 ± 2.3 mM (n=15) in these cells /13/.

From these results we can conclude that this fluorescence technique is an effective and sensitive tool to determine $[Mg^{2+}]_i$ in different biological samples.

### Confocal microscopy experiments

Confocal microscopy and the fluorescent indicator Magnesium Green were used to study the spatial distribution of the intracellular free $Mg^{2+}$ in bovine chromaffin cells. The image obtained with the cells previously loaded with Magnesium Green-AM™ and continuously perfused with Krebs medium (37 °C) is shown in **Fig. 2**. The distribution of the probe inside the nucleus and in the cytoplasm, which is reflected by the probe emission fluorescence intensity observed in the cells' image, depends on the degree of incorporation of the fluorescent probe inside them.

The image shows that the emission fluorescence intensity of the Magnesium Green-$Mg^{2+}$ complex is mainly located in the nucleus of the cells and heterogeneously localised in the cytoplasm mostly around the nucleus, probably in the endoplasmic reticulum. The high fluorescence intensity in the nucleus can be due to a high free $Mg^{2+}$ concentration inside it and/or to an accumulation of the probe in this organelle. It is known that compartmentalization into cellular compartments and binding to proteins can be a problem in the use of fluorescent indicators in cells /65/.

The image of the cells does not have enough resolution to decide if the residual fluorescence from the peripheral part of the cytoplasm comes from the Magnesium Green-$Mg^{2+}$ complex inside the granules or from other organelles. Analysis of **Fig. 2** does not allow one to draw any conclusion about the presence and distribution of free $Mg^{2+}$ in the granules.

It is also possible to observe that in the cell membrane and close to it the fluorescence emission is due to the fluorescence of the uncomplexed probe, indicating that no free $Mg^{2+}$ is located in this part of the cell, which is not surprising since $Mg^{2+}$ is usually bound to the negatively charged binding sites in the membrane (e.g. phospholipids, proteins).

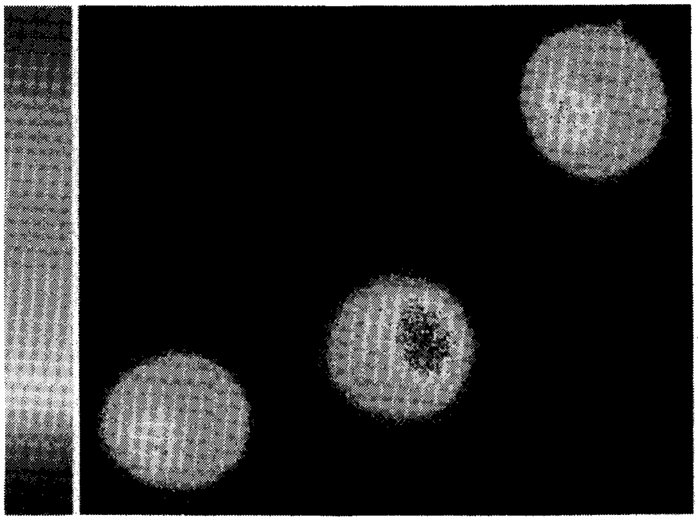

**Fig. 2:** Confocal image of the localization of fluorescent Magnesium Green dye within bovine chromaffin cells. The pseudocolour scale represents the lowest and highest fluorescence signals as dark blue and red, respectively, in arbitrary fluorescence units.

### CONCLUSIONS

In this work the determination of $[Mg^{2+}]_i$ and its spatial localization in bovine chromaffin cells were carried out using three different methods.

$^{31}P$ NMR and fluorescence spectroscopic techniques were used in order to quantify the intracellular free $Mg^{2+}$ in these cells. The method developed by Gupta and his collaborators /48, 49/ to determine the $[Mg^{2+}]_i$ by $^{31}P$ NMR proved to be useless in this cell type, although it has been successfully used in biomolecules /50, 60, 61, 62/ and other cell types /47, 63/. This is due to the particular characteristics of these cells which contain two different pools of ATP, granular and cytosolic pools; the overlap of the $P_\alpha$ NMR resonances of $ATP_{cyt}$ and $ATP_{gran}$ prevented the determination of $[Mg^{2+}]_i$ in the cytosol, while specific $ATP_{gran}$ interactions and the unusually low intragranular pH value and high ionic strength did not allow the quantification of the intragranular free $Mg^{2+}$ using this method.

Fluorescence spectroscopy with the $Mg^{2+}$-sensitive fluorescent probe furaptra proved to be a more accurate and sensitive technique to determine $[Mg^{2+}]_i$ in these cells, giving similar values to the ones obtained for other cell types, as described in the literature.

Confocal microscopy using the $Mg^{2+}$-sensitive fluorescent probe Magnesium-Green proved to be a useful tool to visualize the spatial distribution of $Mg^{2+}$ inside bovine chromaffin cells. However, due to some technical inherent limitations such as probe compartmentalization and insufficient spatial resolution, it was not possible to draw further conclusions about free $Mg^{2+}$ distribution in these cells.

### ACKNOWLEDGEMENTS

The authors acknowledge financial support from Fundação para a Ciência e a Tecnologia (F.C.T.), Portugal (Project POCTI/1999/BCI/36160). C.P. Fonseca and L.P. Montezinho were supported by F.C.T. grants, Praxis XXI/BD/21462/99 and SFRH/BD/3286/2000, respectively.

### REFERENCES

1. R.A. Reinhart, *Arch. Intern. Med* **148**, 2415-2480. (1988).
2. P.W. Flatman, *J. Membr. Biol.* **80**, 1-14 (1984).
3. G.J. Henrotte, in *Magnesium and the Cell*, N.J. Birch (ed.) 177-196, Academic Press, London, 1993.
4. J. Storch, D. Schachter, *Biochim. Biophys. Acta* **812**, 473-484 (1985).
5. A.D. Beavis, K.D. Garlid *J. Biol. Chem.* **262**, 15085-15093 (1987).
6. J. Ishizuka, R.J. Bold, C.M. Townsend, Jr., J.C. Thomson, *Magnesium Res.* 7, 17-22 (1994).
7. K. Kasahara, K. Tasaka, N. Masumoto, T. Nishizaki, J. Mizuki, M. Tahara, A. Miyake, O. Tanizawa, *Biochem. Biophys. Res. Commun.* **197**, 92-99 (1993).
8. F.W. Heaton, in *Metal Ions in Biological Systems: Compendium of Magnesium and its Role in Biology, Nutrition and Physiology*, H. Sigel and A. Sigel (eds.), Chapter 7, Vol. 26, 119-133, M. Dekker, Inc., New York, Basel, 1990.
9. F.W. Heaton, in *Magnesium and the Cell*, N.J. Birch (ed.), 121-136, Academic Press, London, 1993.
10. P.W. Flatman, in *Magnesium and the Cell*, N.J. Birch (ed.), 197-216, Academic Press, London, 1993.
11. C.H. Fry, A.V. Proctor, in *Magnesium and the Cell*, N.J. Birch (ed.), 217-234, Academic Press, London, 1993.
12. J.H. Phillips, Y.P. Allison, *Neuroscience* **2**, 147-152 (1977).
13. G. Pocock, *Mol. Pharmacol.* **23**, 681-697 (1983).
14. M.D. Yago, M. Mañas, J. Singh, *Front. Biosci.* **5**, D602-D618 (2000).
15. H.G. Seiler, in *Metal Ions in Biological Systems: Compendium of Magnesium and its Role in Biology, Nutrition and Physiology*, H. Sigel and A. Sigel (eds.), Chapter 30, Vol. 26, 611-624, M. Dekker, Inc., New York, Basel, 1990.
16. T. Günther, *Magnesium* **5**, 53-59 (1986).
17. B. Raju, E. Murphy, L.A. Levy, R.D. Hall, R.E. London, *Am. J. Physiol.* **256**, C540-C548 (1989).
18. L.A. Blatter, *Pflügers Arch.* **416**, 238-246 (1990).
19. R.E. London, *Ann. Rev. Physiol.* **53**, 241-258 (1991).
20. T. Günther, in *Metal Ions in Biological Systems: Compendium of Magnesium and its Role in Biology, Nutrition and Physiology*, H. Sigel and A. Sigel (eds.), Chapter 11, Vol. 26, 193-213, M. Dekker, Inc., New York, Basel, 1990.
21. P.W. Flatman, *Annu. Rev. Physiol.* **53**, 259-271 (1991).
22. R.K. Gupta, P. Gupta, W. Yushok, Z.B. Rose, *Physiol. Chem. Phys. NMR* **15**, 265-280 (1983).
23. M.J. Kushmerick, P.F. Dillon, R.A. Meyer, T.R. Brown, J.M. Krisanda, H.L. Sweeney, *J. Biol. Chem.* **261**, 14420-14429 (1986).

24. W.R. Adam, D.J. Craik, J.G. Hall, M.M. Kneen, R.M. Wellard, *Magn. Reson. Med.* **12**, 328-338 (1986).
25. H. Degani, A. Shafer, T.A. Victor, A.M. Kaye, *Biochemistry* **23**, 2572-2577 (1984).
26. J.P. Headrick, R.J. Willis, *Magn. Reson. Med.* **12**, 328-337 (1989).
27. E. Murphy, C. Steenbergen, L.A. Levy, B. Raju, R.E. London, *J. Biol. Chem.* **264**, 5622-5627 (1989).
28. E. Heinonen, K.E. Akerman, *Biochim. Biophys. Acta* **898**, 331-337 (1987).
29. B.E. Corkey, J. Duszynski, T.L. Rich, B. Matschinsky, J.R. Williamson, *J. Biol. Chem.* **261**, 2567- 2574 (1986).
30. E. Murphy, C.C. Freudenrich, M. Lieberman, *Annu. Rev. Physiol.* **53**, 273-287 (1991).
31. C. Cheng, I.J. Reynolds, *Neuroscience* **95**, 973-979 (2000).
32. B.B. Jacques, R. Sunita, I.R. Ian, *Neuron* **11**, 751-757 (1993).
33. D. Veloso, R. Guynn, M. Oskarsson, R.L. Veech, *J. Biol. Chem.* **248**, 4811-4819 (1973).
34. C.R. Malloy, C.C. Cunninham, G.K. Radda, *Biochim. Biophys. Acta* **885**, 1-11 (1986).
35. N.M. Ramadan, H. Halvorsen, A. Vande-Linde, S.R. Levine, J.A. Helpern, K.M.A. Welch, *Headache* **29**, 416-419 (1989).
36. R. Vink, J.K. McIntosh, P. Demediuk, M.W. Weiner, A.I. Faden, *J. Biol. Chem.* **263**, 757-761 (1988).
37. J.P. Sheehan, *Magnesium Trace Elem.* **10**, 215-219 (1991).
38. E. Gueux, C. Cubizolles, L. Bussiere, A. Mazur, Y. Rayssiguier, *Lipids* **28**, 573-575 (1993).
39. T. Fujita, T. Kano, S. Kobayashi, in *The Paraneuron*, T. Fujita, T. Kano and S. Kobayashi (eds.), Chapter 12, 135-144, Springer-Verlag, Tokio, Japan, 1988.
40. K. Unsicker, *J. Anat.* **183**, 207-221 (1993).
41. R.B. Johnson, S.E. Carty, A. Scarpa, *Ann. N. Y. Acad. Sci.* **456**, 254-267 (1985).
42. J.H. Phillips, in *Stimulus-Secretion Coupling in Chromaffin Cells*, K. Rosenheck and P.I. Lelkes (eds.), Vol. I, Chapter 3, 55-85, CRC Press, Boca Raton, Florida, 1987.
43. K.W. Brocklehurst, H.B. Pollard, in *Peptide Hormones: a Practical Approach,* K. Siddle and J. Hutton (eds.), 233-255, IRL Press, Oxford, 1990.
44. S.P. Wilson, *J. Neuroscience Methods* **19**, 163-171 (1987).
45. M.A. Moro, M.G. L6pez, L. Gandía, P. Michelena, A.G. García, *Analytical Biochemistry* **185**, 243-248 (1990).
46. J. Nikolakopoulos, C. Zachariah, D.M. Freitas, E.B. Stubbs, Jr., R. Ramasamy, M.M.C.A. Castro, C.F.G.C. Geraldes, *J. Neurochem.* **71**,1676-1684 (1998).
47. L. Amari, B. Layden, J. Nikolakopoulos, Q. Rong, D.M. Freitas, G. Baltazar, M.M.C.A. Castro, C.F.G.C. Geraldes, *Biophys. J.* **76**, 2934-2942 (1999).
48. R.K. Gupta, J.L. Benovic, Z.B. Rose, *J. Biol. Chem.* **253**, 6165-6171 (1978).
49. R.K.Gupta, in *NMR in Living Systems*, T.Axenrod and G.Ceccarelli (eds.), 335-345, Reidel Publishing Company, Dordrecht, Netherlands, and references therein (NATO ASI Series, C164), 1986.
50. A. Abraha, D.M. Freitas, M.M.C.A. Castro, C.F.G.C. Geraldes, *J. Inorg. Biochem.* **42**, 191-198 (1991).
51. S. Iotti, C. Frassineti, L. Alderighi, A. Sabatani, A. Vacca, B. Barbiroli, *NMR Biomed.* **9**, 24-32 (1996).
52. R.K. Gupta, J.L. Benovic, *Biochem. Biophys. Res. Commun.* **84**, 130-137 (1978).
53. E. Murphy, C.C. Freudenrich, L.A. Levy, R.E. London, M. Lieberman, *Proc. Natl. Acad. Sci. USA* **86**, 2981-2984 (1989).

54. C.P. Fonseca, L.P. Montezinho, G. Baltazar, B. Layden, D.M. Freitas, C.F.G.C. Geraldes, M.M.C.A. Castro, *Metal Based Drugs* 7, 357-364 (2000).
55. R.P. Haugland, in *Handbook of Fluorescent Probes and Research Chemicals* - 8th edition, M.T.Z. Spence (ed.), Chapter 20, Section 20.6, 2002.
56. J.J. Corcoran, M. Korner, B. Caughey, N. Kirshner, *J. Neurochem.* **47**, 945-952 (1986).
57. G.R. Painter, E.J. Diliberto, Jr., J. Knoth, *Proc. Natl. Acad. Sci. USA* **86**, 2239-2242 (1989).
58. D. Njus, P.A. Sehr, G.K. Radda, G.A. Ritchie, P.J. Seeley, *Biochemistry* 17, 4337-4343 (1978).
59. M.A. Foster, in *Magnetic Resonance in Medicine and Biology*, Chapter 6, 125, Pergamon Press, Oxford, 1984.
60. L. Amari, B. Layden, Q. Rong, C.F.G.C. Geraldes, D.M. Freitas, *Anal. Biochem.* **272**, 1-7 (1999).
61. D.M. Freitas, L. Amari, C. Srinivasan, Q. Rong, R. Ramasamy, A. Abraha, C.F.G.C. Geraldes, M.K. Boyd, *Biochemistry* **33**, 4101-4110 (1994).
62. Q. Rong, D.M. Freitas, C.F.G.C. Geraldes, *Lithium* **3**, 213-220 (1992).
63. B. Layden, C.P. Fonseca, N. Minadeo, H. Abdullahi, M.M.C.A. Castro, C.F.G.C. Geraldes, D.M. Freitas, in *Lithium-50 Years: Recent Advances in Biology and Medicine*, K.C. Lukas, R.W. Becker and V.S. Gallichio (eds.), 45-62, Weidner Publishing, Cheshire, Connecticut, USA, 1999.
64. O. Kaplan, P. Aebersold, J.S. Cohen, *FEBS Lett.* **258**, 55-58 (1989).
65. J. Pesco, J.M. Salmon, J. Vigo, P. Viallet, *Anal. Biochem.* **290**, 221-231 (2001).

